# Biocatalytic potential of laccase-like multicopper oxidases from *Aspergillus niger*

**DOI:** 10.1186/1475-2859-11-165

**Published:** 2012-12-27

**Authors:** Juan Antonio Tamayo-Ramos, Willem JH van Berkel, Leo H de Graaff

**Affiliations:** 1 Microbial Systems Biology, Laboratory of Systems and Synthetic Biology, Wageningen University, Dreijenplein 10, Wageningen, 6703 HB, The Netherlands; 2 Laboratory of Biochemistry, Wageningen University, Dreijenlaan 3, Wageningen, 6703 HA, The Netherlands

**Keywords:** *Aspergillus*, Multicopper oxidase, Laccase, Bioremediation, Decolorization

## Abstract

**Background:**

Laccase-like multicopper oxidases have been reported in several *Aspergillus* species but they remain uncharacterized. The biocatalytic potential of the *Aspergillus niger* fungal pigment multicopper oxidases McoA and McoB and ascomycete laccase McoG was investigated.

**Results:**

The laccase-like multicopper oxidases McoA, McoB and McoG from the commonly used cell factory *Aspergillus niger* were homologously expressed, purified and analyzed for their biocatalytic potential. All three recombinant enzymes were monomers with apparent molecular masses ranging from 80 to 110 kDa. McoA and McoG resulted to be blue, whereas McoB was yellow. The newly obtained oxidases displayed strongly different activities towards aromatic compounds and synthetic dyes. McoB exhibited high catalytic efficiency with *N,N*-dimethyl-*p*-phenylenediamine (DMPPDA) and 2,2-azino-di(3-ethylbenzthiazoline) sulfonic acid (ABTS), and appeared to be a promising biocatalyst. Besides oxidizing a variety of phenolic compounds, McoB catalyzed successfully the decolorization and detoxification of the widely used textile dye malachite green.

**Conclusions:**

The *A. niger* McoA, McoB, and McoG enzymes showed clearly different catalytic properties. Yellow McoB showed broad substrate specificity, catalyzing the oxidation of several phenolic compounds commonly present in different industrial effluents. It also harbored high decolorization and detoxification activity with the synthetic dye malachite green, showing to have an interesting potential as a new industrial biocatalyst.

## Background

Multicopper oxidases (MCOs) form a family of redox enzymes that catalyze the reduction of molecular oxygen into water by a four-electron transfer process. It includes laccases (EC 1.10.3.2), ascorbate oxidases (EC 1.10.3.3), bilirubin oxidases (EC 1.3.3.5) and ferroxidases (EC 1.16.3.1), which are key enzymes in many biological processes of prokaryotic and eukaryotic organisms [1,2]. In fungi, complex MCO gene families exist, possibly due to the variety of functions they accomplish. Fungal MCOs are involved in delignification, morphogenesis, pigment formation, pathogenesis, competitor interactions and transport of metal ions [2,3]. Their ability to react with a variety of aromatic compounds, by producing just water as a by-product, makes them interesting green biocatalysts [2,3]. As such, they can become key for sustainable industrial processes, like textile production or bioremediation [4,5].

The majority of fungal MCOs are distributed, according to Hoegger *et al*. [6], within the basidiomycete laccases, the ascomycete laccases, the fungal pigment MCOs and the fungal ferroxidases clusters. Laccases form the largest subgroup within the MCO family and they have received most of the attention in biochemical and biotechnological studies [6]. In particular, basidiomycete laccases of several *Trametes* and *Pleurotus* species, amongst others, have been well characterized [2].

Fungal pigment MCOs, mainly found in ascomycetes [7], have been reported in several *Aspergillus* species: *A. nidulans* LccD, TilA and YA [8-10]; *A. fumigatus* Abr2 [11]; and *A. niger* McoA, McoB and McoC [12]. Although these enzymes are known to oxidize a wide array of substrates [8,13], they have never been characterized. Therefore no information is available about their molecular properties or substrate specificities. *Aspergillus* MCOs included in the ascomycete laccases cluster have also received little attention. A significant number of these enzymes, including: *A. nidulans* LccA, LccB and LccC [8]; and *A. niger* McoD, McoF, McoG, McoI, McoJ and McoM [12], remain uncharacterized. Interestingly, *A. niger* MCOs (both, the ones that belong to the fungal pigment MCO cluster, and to the ascomycete laccase cluster) have a low similarity to laccases included in the basidiomycete laccases cluster (around 25% identical). They also differ from the few deeply characterized ascomycete laccases (i.e. around 25-30% identical to *Melanocarpus albomyces* laccase, MaL). Thus, to obtain insight into the possible biotechnological potential of this particular group of MCOs, more knowledge about their catalytic properties is required.

The activity patterns observed in plate assays of ten *A. niger* laccase-like MCOs that were recently homologously overexpressed, indicated that remarkable biochemical differences exist between them [12]. Here we address the biocatalytic potential of three *A. niger* laccase-like MCOs: two fungal pigment MCOs (McoA and McoB), and one MCO belonging to the ascomycete laccase subfamily (McoG). Their ability to oxidize an array of aromatic compounds and decolorize different dyes was evaluated.

## Results and discussion

### Homologous expression, purification and molecular properties of *A. niger* MCOs

**Figure 1:**
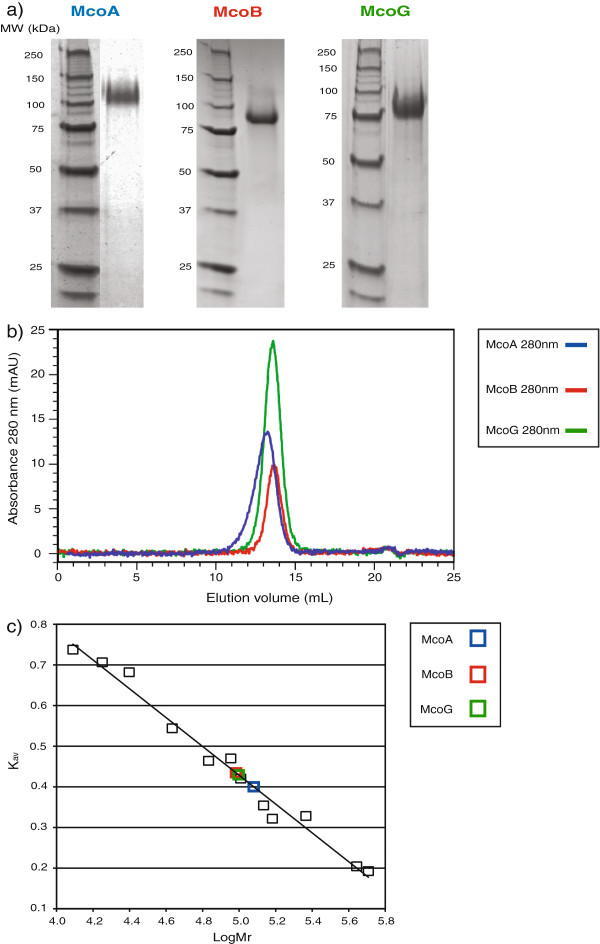
**SDS-PAGE (a), elution profiles from a Superdex 200 HR 10/30 column (b) of McoA, McoB and McoG. **Reference proteins used to calibrate the Superdex 200 HR 10/30 column and calculate the McoA, McoB and McoG molecular mass **(c)**. The gel filtration calibration was performed with the following reference proteins: cytochrome c (12 kDa), myoglobin (18 kDa), α-chymotrypsin (25 kDa), ovalbumin (43 kDa), bovine serum albumin (68 and 136 kDa), 4-hydroxybenzoate 3-hydroxylase (90 kDa), lipoamide dehydrogenase (102 kDa), phenol 2-hydroxylase (152 kDa), catalase (232 kDa), ferritin (440 kDa) and vanillyl-alcohol oxidase (510 kDa). McoA, McoB and McoG were also included in the plot of K_av_ versus LogM_r_.

In order to bring the first insights about the molecular properties and biotechnological potential of fungal pigment MCOs, McoA, McoB and McoC were selected for their purification and characterization. McoG was chosen to be investigated in this study as well, because it showed (together with McoB) the broadest substrate specificity in plate activity assays [12]. Only McoA, McoB and McoG could be purified in sufficient amounts and with enough quality to continue with their characterization. The three recombinant laccase-like MCOs were purified to apparent homogeneity from 24 h culture supernatants (see Material and Methods). Their apparent molecular masses, observed by SDS-PAGE, were ~110 kDa for McoA, ~88 kDa for McoB and ~80 kDa for McoG (Figure 1a), being in all cases higher than the theoretical expected value (~64 kDa for McoA, ~63 kDa for McoB and ~65 kDa for McoG). This difference in size may originate from post-translational protein processing, such as glycosylation. Indeed, analysis of the three amino acid sequences with NetNGlyc 1.0 and GPP Prediction Servers revealed the presence of several potential N-glycosylation sites, being more predominant in McoA (data not shown). Gel filtration, using a calibrated Superdex 200 column, was performed in order to determine the size and subunit composition of the three enzymes. A single peak was observed for the native form of each MCO, with a relative molecular mass estimated to be: ~120 kDa for McoA, ~96 kDa for McoB and ~99 kDa for McoG (Figure 1b and 1c). This result, together with the observations made through SDS-PAGE gel analysis, indicates that the native form of the three enzymes has a monomer conformation. 

**Figure 2:**
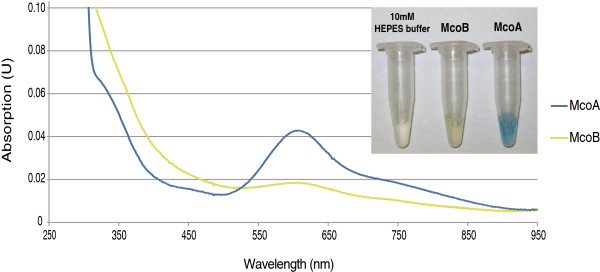
**Absorption spectra of McoA and McoB (~1 mg/mL). **Embedded in the graph, McoA and McoB color solutions (10-15 mg/mL) can be observed and compared with 10 mM HEPES buffer (pH 7.0).

Concentrated enzyme solutions (10-15 mg/mL) of McoA and McoG displayed a blue color, whereas McoB solution was yellowish (Figure 2). In fact, when comparing the absorption spectra (300-950 nm) of McoA and McoB, it could be observed that McoB absorbance at 610 nm was relatively low (Figure 2). On the other hand, McoB showed an increased absorbance at 420-430 nm when compared with McoA. The available information about the origin of the yellow color of some characterized MCOs is still very limited [14-18]. Thus, in order to better understand this phenomenon, more experimental data are needed. 

### Kinetic parameters

The kinetic parameters of the three enzymes were determined    using     *N,N*-dimethyl-*p*-phenylenediamine (DMPPDA) as substrate and Michaelis-Menten modeling. For McoB and McoG, kinetic constants were also determined with 2,2-azino-di(3-ethylbenzthiazoline) sulfonic acid (ABTS) (Table 1). McoA could not oxidize ABTS, as reported for LccD from *A. nidulans*[8] and the laccase recently characterized from *Bacillus sp*. ADR [19]. McoB showed a much higher affinity and activity with DMPPDA than McoA and McoG. Remarkably, McoB and McoG activity was reduced at higher DMPPDA concentrations, presumably due to substrate inhibition (Table 1). This inhibition was particularly strong in McoG, as the *K*_*i*_ is close to the apparent *K*_*m*_. McoB has more affinity for ABTS than McoG, showing a similar *K*_*m*_ as *Pleurotus ostreatus* POXA1b laccase [20]. The specific activity of both *A. niger* enzymes for ABTS is similar to that reported for several ligninolytic fungi laccases [14] and higher to the one reported for *Melanocarpus albomyces* laccase [21]. 

**Table 1:** Steady-state kinetic parameters of McoA, McoB and McoG with DMPPDA and ABTS

	**DMPPDA**			**ABTS**		
	***K*_***m***_*(mM)***	***V*_***max***_*(Δ*Α_**550**_*/min mg)***	***K*_***i***_*(mM)***	***K*_***m***_*(mM)***	***V*_***max***_*(Δ*Α_**420**_*/min mg)***	***K*_***i***_*(mM)***
**McoA**	3.6 ± 0.2	4.6 × 10^2^ ± 8	n.d.	n.d.	n.d.	n.d.
**McoB**	0.4 ± 0.1	7.5 × 10^3^ ± 6.0 × 10^2^	22.4 ± 6.4	0.5 ± 0.1	2.9 × 10^2^ ± 4	n.d.
**McoG**	1.8 ± 0.3	4.4 × 10^2^ ± 40	4.6 ± 0.7	5.6 ± 1.0	2.5 × 10^2^ ± 16	n.d.

n.d. = not detectable.

### Effect of pH and temperature on *A. niger* MCOs activity

**Figure 3:**
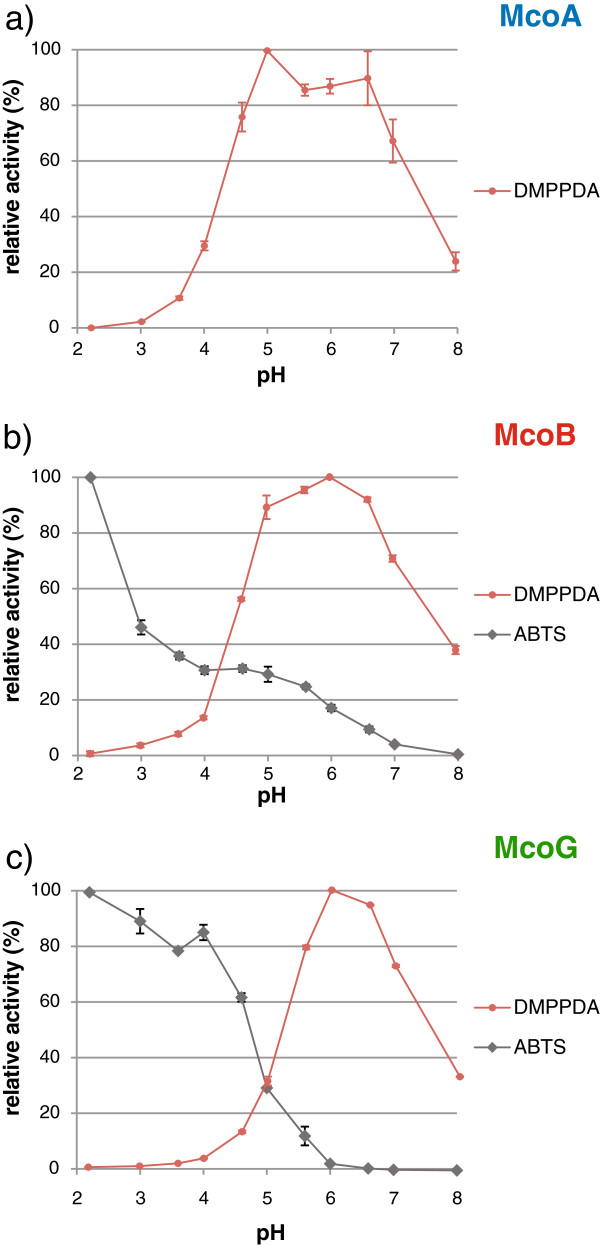
**Effect of pH in McoA (a), McoB (b) and McoG (c) activity. **The initial rate of enzyme activity was measured with DMPPDA and ABTS substrates in pH range 2.2 to 8.0.

The pH optima of the *A. niger* MCOs for the oxidation of DMPPDA were similar to that of other *Aspergillus* extracellular enzymes. Initial rate measurements revealed that McoA activity was highest at pH 5.0, whereas pH 6.0 was the optimum for McoB and McoG (Figure 3). McoA and McoB displayed a broader optimal range for catalyzing the oxidation of DMPPDA than McoG, showing at least 80% of their optimal activity in a wide pH range. McoB and McoG oxidized ABTS in acidic conditions with an optimum pH of 2.2 (Figure 3). The higher rate of ABTS oxidation at low pH was already described for other fungal laccases [22]. Two opposite effects: the difference in redox potential between the substrate and the T1 copper (that could increase oxidation of the substrate at high pH values) and the laccase activity inhibition that results from hydroxide anion (OH^-^) binding to the T2/T3 coppers, could play an important role in determining the optimal pH of these enzymes [23]. Nevertheless, by continuously monitoring the delta absorbance (420 nm) during the incubation of both enzymes with ABTS, it could be observed that in the pH range of 2.2 to 4.0, the activities of McoB and McoG decreased faster in time than at higher pH (data not shown). The remaining activity rates of both enzymes after 30 min, when compared to their initial values, were: 11% to 22% for McoB and 7% to 10% for McoG at the pH range of 2.2 to 4.0, whereas at pH 6.0 McoB kept 93% of its initial activity rate and McoG 100%. This observation suggests that both enzymes could be less kinetically stable or more susceptible to product inhibition at lower pH. The fact that the activity rate of McoB after 30 min at pH 4.0 was two times lower than at pH 4.6 while their initial activity rates were almost the same, supports this hypothesis. 

**Figure 4:**
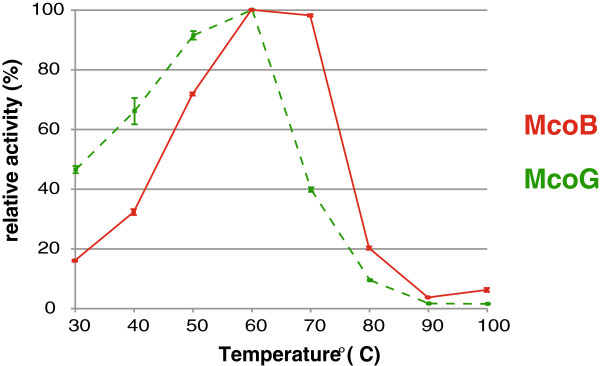
**Effect of temperature in McoB (red line) and McoG (green dashed line) activity. **The enzyme activity was measured, using ABTS as a substrate, in the range 20 to 100°C, using 100 mM sodium acetate buffer (pH 5.0).

The temperature optimum of the *A. niger* MCOs was determined only for the reaction with ABTS, as DMPPDA is unstable at high temperature. Also the 4-amino-2,6-dibromophenol/3,5-dimethylaniline (ADBP/DMA) assay did not produce reliable results at high temperature, therefore McoA optimal temperature was not determined, as no other substrate with a reproducible assay for testing McoA activity (in a wide range of temperatures) is known so far. Optimal temperature for McoB and McoG catalysis was 60°C (Figure 4). The data obtained indicate that McoB retains at least 80% of its activity from 50 to 75°C, whereas McoG shows more than 80% of its activity between 45 and 62°C. 

### Effect of potential inhibitors on *A. niger* MCOs activity

The effect of ten compounds on the activity of the purified MCOs was tested (Table 2). McoA, McoB and McoG were strongly inhibited by 1 mM NaN_3_ as reported for many laccases [24]. Different concentrations of CuSO_4_ (0.1, 0.5, 1.0 and 2.5 mM) also highly inhibited McoG activity, whereas McoA and McoB inhibition was more moderate, and similar between both enzymes. The presence of additional copper ions to the ones present in the catalytic site of MCOs have been reported to be an inhibition factor [25]. Organic solvents methanol, ethanol and acetone, at a final concentration of 50% (v/v), also caused a severe inhibition (between 54 and 89%) of the three enzymes. In contrast, salts 50 mM CaCl_2_, 50 mM MgCl_2_, and 100 mM NaCl produced a lower inhibitory effect, particularly in McoB, which retained high levels of activity (from 76 to 91%). In the presence of 50 mM EDTA, partial inhibition was observed for McoA and McoB, whereas activity of McoG was two-fold increased. EDTA might activate McoG by chelating metal ions, different than copper, that could be bound to the enzyme causing a partial inhibition of its activity. SDS (1 mM) did not produce any inhibitory effect. In contrast, it slightly stimulated *A. niger* MCOs activity, as reported for other phenol oxidases [26]. The reduction capacity of the reaction product by the inhibitors was not determined in this study, hence it cannot be discarded that this phenomenon occurs with some of the compounds tested. Therefore, additional substrates or oxygen consumption measurements could be assayed in order to confirm these results. 

**Table 2:** Effect of metal salts and inhibitors on McoA, McoB and McoG activity

**Inhibitor**	**Residual activity (%)**		
	**McoA**	**McoB**	**McoG**
NaN_3_ (1 mM)	5.0 ± 0.3	20.0 ± 1.3	16.6 ± 4.2
EDTA (50 mM)	93.8 ± 1.8	82.9 ± 9.8	202.8 ± 33.9
CaCl_2_ (50 mM)	63.7 ± 0.4	76.4 ± 4.2	53.3 ± 8.6
MgCl_2_ (50 mM)	81.3 ± 0.1	91.0 ± 0.3	78.2 ± 0.5
NaCl (100 mM)	72.5 ± 4.1	83.8 ± 4.2	62.0 ± 9.5
CuSO_4_ (0.1 mM)	83.0 ± 10.2	73.1 ± 10.9	14.2 ± 6.5
CuSO_4_ (0.5 mM)	51.3 ± 8.1	41.9 ± 1.6	2.9 ± 2.0
CuSO_4_ (1 mM)	36.2 ± 6.7	43.0 ± 3.1	n.d.
CuSO_4_ (2.5 mM)	31.6 ± 6.4	36.2 ± 3.8	n.d.
SDS (1 mM)	109.0 ± 1.0	139.0 ± 10.1	143.7 ± 27.0
MetOH (50%)	46.1 ± 4.4	29.8 ± 1.3	21.9 ± 5.5
EtOH (50%)	10.8 ± 1.2	34.6 ± 1.1	13.9 ± 4.0
Acetone (50%)	28.8 ± 0.4	46.1 ± 4.2	24.1 ± 1.6

The 100% specific activity of McoA, McoB and McoG was 72.3±1.3 U/mg, 236.1±12.6 U/mg and 57.5±10.6 U/mg, respectively. n.d. = not detectable.

### Biocatalytic potential with natural and synthetic substrates

To gain more insight into the substrate specificity of McoA, McoB and McoG, their specific activities with a variety of aromatic compounds were determined by measuring oxygen consumption (Table 3). All three enzymes catalyzed the oxidation of ADBP, phenol and hydroquinone. McoA and McoB were active with ferulic acid and McoB also oxidized 2,6-dimethoxyphenol, vanillic and syringic acid. Only McoG reacted with the non-phenolic cinnamic acid, and was also active with 2,6-dimethoxyphenol. None of the three enzymes was active with *p*-coumaric acid and vanillin under the tested conditions. Compounds like hydroquinone, ferulic acid, vanillic acid and syringic acid are generated during lignin decomposition [27,28], and, together with phenol, they are present in the effluent of different industries, like olive oil mill or pulp and paper among others [29,30]. Therefore McoB might be a good candidate to be used in pre-treatment processes of these types of wastewaters. 

**Table 3:** Substrate specificity of McoA, McoB and McoG

**Substrate (1 mM)**	**McoA (U/mg)**	**McoB (U/mg)**	**McoG (U/mg)**
ADBP	7.1 × 10^3^ ± 1.6 × 10^2^	13.0 × 10^3^ ± 5.6 × 10^2^	75.6 × 10^3^ ± 2.3 × 10^3^
Phenol	1.7 × 10^3^ ± 4.1 × 10^2^	3.1 × 10^3^ ± 7.9 × 10^2^	2.9 × 10^3^ ± 3.1 × 10^2^
2,6-dimethoxyphenol	n.d.	7.0 × 10^3^ ± 4.1 × 10^2^	4.9 × 10^3^ ± 2.9 × 10^3^
Hydroquinone	6.6 × 10^2^ ± 3.2 × 10^2^	6.1 × 10^3^ ± 4.4 × 10^2^	4.5 × 10^3^ ± 1.2 × 10^2^
Cinnamic acid	n.d.	n.d.	7.3 × 10^2^ ± 5.2 × 10^2^
Vanillin	n.d.	n.d.	n.d.
*p*-Coumaric acid	n.d.	n.d.	n.d.
Vanillic acid	n.d.	2.3 × 10^2^ ± 20	n.d.
Ferulic acid	3.9 × 10^2^ ± 1.1 × 10^2^	3.1 × 10^3^ ± 1.1 × 10^2^	n.d.
Syringic acid	n.d.	1.6 × 10^3^ ± 1.1 × 10^2^	n.d.

n.d. = not detectable.

The inability of McoA to oxidize 2,6-dimethoxyphenol (as observed before) nor ABTS is a remarkable result, as a significant number of reported laccases react with these two common substrates [24]. Nevertheless, as discussed in the “Kinetic parameters” section, other laccases from eukaryotic and prokaryotic sources are not active with ABTS as well. Also, other laccases have been reported to be inactive with laccase model substrates. For instance, the *Agaricus bisporus* laccase [31] is unable to convert syringaldazine and EpoA, from *Streptomyces griseus*, does not oxidize syringaldazine and guaiacol [32]. The range of substrates oxidized varies from one laccase to another [33]. It has been suggested that differences in substrate access to the T1 copper site of laccases could imply different substrate affinities [34]. In order to study and understand if this factor could have influence in the narrow range of substrate specificity of McoA, the availability of its three-dimensional structure would be desirable.

### Dye decolorization

**Figure 5:**
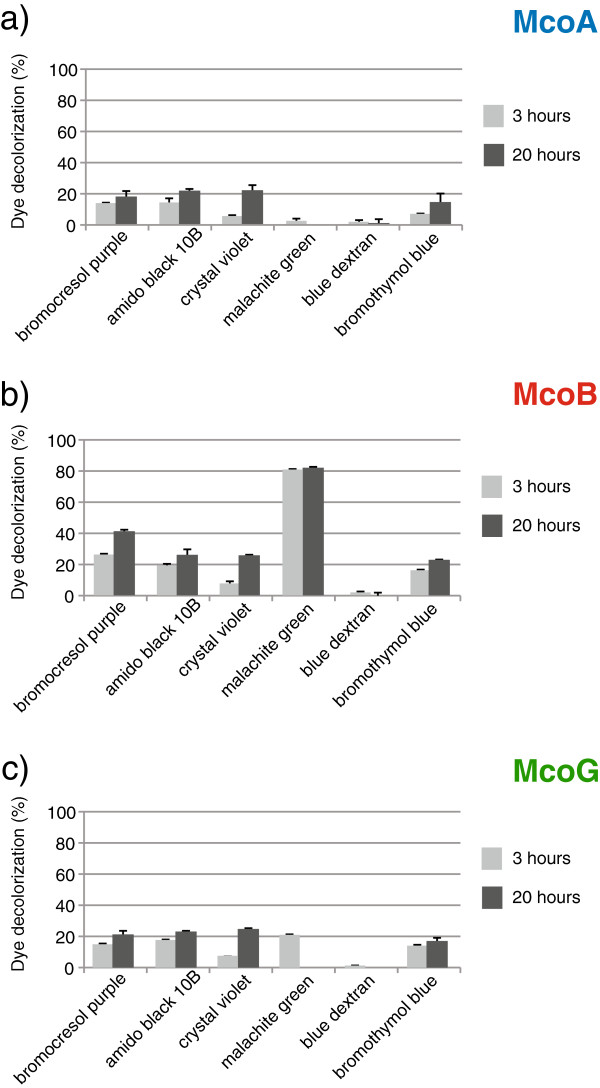
**Decolorization of different dyes (50 mg/L) by McoA (a), McoB (b) and McoG (c). **Purified enzymes were used to decolorize bromocresol purple, amido black 10B, crystal violet, malachite green, blue dextran and bromothymol blue.

The *A. niger* recombinant MCOs were able to oxidize different synthetic dyes (50 mg/L). Degree of decolorization, after 3 and 20 h of incubation at 55°C, was variable in each case (Figure 5). The three enzymes were able to decolorize bromocresol purple, amido black 10B, crystal violet and bromothymol blue. McoB and McoG also reacted with malachite green, whereas none of them was able to decolorize blue dextran. McoA and McoG decolorized the different dyes up to ~20%. McoB showed the best decolorizing ability with bromocresol purple (41% of decolorization after 20 h) and in particular with malachite green (83% decolorization after 20 h). 

**Figure 6:**
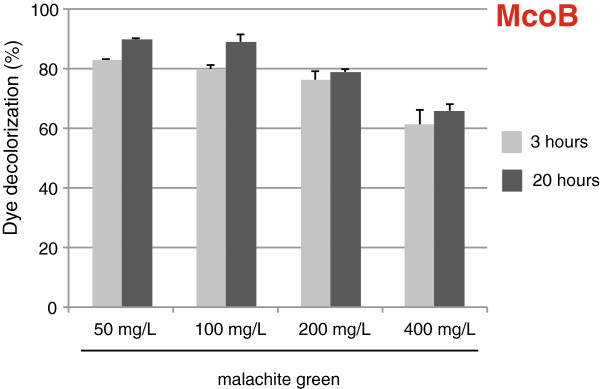
Decolorization of different concentrations (50 mg/L, 100 mg/L, 200 mg/L and 400 mg/L) of malachite green by McoB.

Environmental pollution caused by malachite green (MG) is a serious problem, as this dye has carcinogenic and mutagenic properties, is hardly biodegradable and still widely used by different industries [35,36]. To gain further insight in the ability of McoB to decolorize MG, higher concentrations of this compound were used. Figure 6 illustrates that McoB shows good decolorization capacity when the dye is present at 50 mg/L or 100 mg/L. In both cases 80% of decolorization occurs already after 3 h, and reaches 90% after 20 h. In addition, McoB decolorized around 80% malachite green in a 200 mg/L solution, and around 65% in a 400 mg/L solution. 

The MG decolorization ability of McoB, when the dye is present at a final concentration of 50 mg/L, is similar to the one reported recently from a new laccase from *Trametes* sp. 48424 [37], and better than that of the laccase from the white-rot fungus *Ganoderma lucidum*[38]. The direct decolorization of MG during the cultivation of several basidiomycete and non-basidiomycete fungi has been also investigated, but the reported efficiencies in similar conditions (e.g. incubation time, MG concentration) were lower than the ones achieved by means of using the purified enzymes [39-42]. In addition, McoB was also able to oxidize higher concentrations of MG. Similar results, have not been reported for other fungal laccases, although recently the *Pseudomonas* sp. strain DY1, grown in the presence of the dye, has been shown to be a very efficient tool for MG degradation, in concentration ranges from 100 mg/L to 1000 mg/L [35]. MG successful degradation (50 mg/L) by other bacterial species has been also reported [43,44]. However, in none of the cases the enzymes involved in the biodegradation process were identified. The results obtained in the present study suggest that McoB could be used in bioremediation processes of this compound. To confirm this, the toxicity of MG transformation compounds should be assessed, as its biodegradation pathway varies depending on the biological treatment, and thus the generated intermediates and final products [35,38]. The fact that decolorization of MG was achieved with no need of mediators would mean an additional advantage for the use of McoB, as synthetic mediators may be expensive, toxic, and inhibit the enzyme activity at higher concentrations [45,46]. Nevertheless, natural mediators have also been shown to effectively enhance the transformation of MG in combination with a fungal laccase [38].

### Fungicide activity of MG and its transformation products

**Figure 7:**
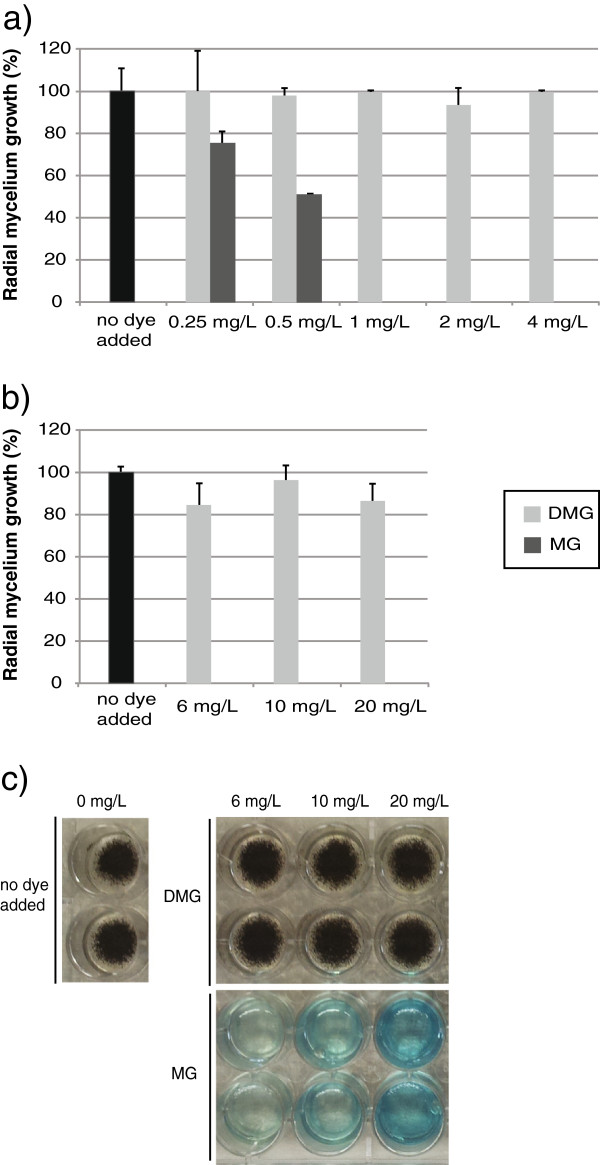
***A. niger *radial growth in complete medium agar plates in the presence of 0.25 mg/L, 0.5 mg/L, 1 mg/L, 2 mg/L and 4 mg/L of malachite green or malachite green decolorized with McoB for 3 h (a); and in the presence of 6 mg/L, 10 mg/L and 20 mg/L of malachite green or malachite green decolorized with McoB for 20 h (b and c). **The radial mycelium growth was measured 24 h after inoculation. The picture illustrates the appearance of *A. niger* N593 strain, grown in complete medium in the presence of different concentrations of MG or DMG, 72 h after inoculation. MG= malachite green; DMG= decolorized malachite green.

Malachite green toxicity spectrum is wide, affecting microorganisms (including fungi) and higher eukaryotes [46]. *A. niger* N593 strain was chosen as a model to evaluate the toxicity of MG transformation products after incubation of the dye with McoB. Initially, *A. niger* was grown in agar plates containing 0.25 mg/L, 0.5 mg/L, 1 mg/L, 2 mg/L and 4 mg/L of MG previously treated with McoB (called hereafter DMG) or with 10 mM HEPES buffer pH 7.0 (used as a non detoxification control) for 3 h. A MG concentration of 0.25 mg/L affected significantly the radial growth of *A. niger*, whereas 1 mg/L initially inhibited it. However, with the latter MG concentration, a poor growth was observed after 72 h of *A. niger* incubation. MG concentrations of 2 and 4 mg/L completely inhibited *A. niger* growth. On the other hand, equal concentrations of DMG did not affect *A. niger* growth (Figure 7a). 

The toxicity of higher concentrations (6 mg/L, 10 mg/L and 20 mg/L) of MG and DMG, obtained by incubating MG with McoB for 20 h was also tested. As expected, MG completely inhibited *A. niger* growth, whereas a negative effect of DMG in the radial growth of *A. niger* was hardly observed (Figure 7b). Furthermore, the radial growth and the mycelium appeareance 72 h after inoculation in complete medium with DMG was not different to the one observed in the control condition, where no dye was added (Figure 7c). These results show that the compounds generated after MG decolorization by McoB have a lower toxic effect in *A. niger* when compared with the one produced by the untreated dye. Therefore, McoB could be a good candidate to be used in detoxification processes of MG. The ability of McoB to detoxify MG without the need of redox mediators is an advantage over other MG detoxification processes reported [38,46].

The different biocatalytic properties of the *A. niger* MCOs determined in this study are remarkable. McoA and McoB, showing 56% of sequence identity, are severely divergent in their biochemical features. The potential applications in wastewaters pre-treatment processes and dye decolorization, along with the fact that yellow MCOs are far less well characterized than their blue counterparts, makes more challenging to continue with a deeper characterization of McoB. In this sense, it would be interesting to test its suitability for applications related to the food industry, as *A. niger* is a safe production organism and many of its enzymes are considered generally recognized as safe (GRAS) by the United States Food and Drug Administration [47]. Also, the possibility to use industrial *A. niger* strains to overproduce the native form of McoB could overcome issues related to production yields and stability [48], that can occur during heterologous expression of recombinant proteins, and are less expected in homologous expression systems [6]. Interestingly, the production yields of this enzyme have been recently optimized, together with those of other *A. niger* MCOs [49]. By improving the performance of the glucoamylase expression system, *A. niger* strains able to accumulate extracellular McoB up to 42% of the total secreted protein were generated [49].

## Conclusions

This study reports the first biochemical characterization of *A. niger* MCOs. The purified McoA, McoB, and McoG enzymes showed clearly different substrate specificities. Yellow McoB turned out to be the most efficient biocatalyst, showing broad substrate specificity and high decolorization activity with the synthetic dye malachite green. The lower fungicide activity of decolorized MG suggests that McoB can be an interesting biocatalyst for bioremediation processes involving this dye.

## Methods

### Strains, media and culture conditions

*A. niger* N593 strains expressing recombinant McoA, McoB and McoG, were used as cell factories to produce the three enzymes [12]. Complete medium plates were used for spores preparation and fungicide activity tests. Minimal medium [50] liquid cultures (containing 50 mM of maltose and 0.1 mM of CuSO_4_) were used for MCOs production. Liquid cultures were performed at 30°C and 250 rpm in an orbital shaker, in 2 L erlenmeyer flasks containing 800 mL of minimal medium inoculated with 1 × 10^6^ spores/mL.

### Purification of *A. niger* MCOs

All MCO proteins were purified from filtered supernatants of 24 h cultures. Ammonium sulfate was applied at 100% saturation in 3 L of culture supernatant containing McoA. After centrifugation, the recovered pellet was dissolved in 50 mL of 20 mM Tris-HCl buffer (pH 7.5). The resulting solution was dialyzed against 20 mM Tris-HCl buffer (pH 7.5) and then concentrated using an Amicon Ultra-15 Centrifugal Filter device. The resulting solution was applied to a Resource Q 1mL column (GE Healthcare) installed in an Äkta purifier FPLC system (GE Healthcare). A linear gradient of NaCl from 0 to 1 M in 20 mM Tris-HCl buffer (pH 7.5) was performed in a total volume of 200 mL, at a flow rate of 1 mL/min.

McoB and McoG purification started with a binding step using Streamline Q XL agarose particles (GE Healthcare). Prior to the binding step, 1.5 L of culture supernatants containing McoB and McoG were diluted five times in de-mineralized water. 50 mL of Streamline Q XL resin was added to each solution, and stirred during 3 h at 4°C. Proteins were eluted from the resin with 50 mL of a 1 M NaCl solution in 20 mM Tris-HCl buffer (pH 7.5). McoB and McoG solutions were concentrated in an Amicon Ultra-15 Centrifugal Filter device and the NaCl concentration was severely reduced through several washing steps with 20 mM Tris-HCl buffer (pH 7.5). McoB and McoG were further purified on Resource Q, using the same protocol as described for McoA. Purified enzymes were stored at -80°C in 10 mM HEPES buffer (pH 7.0).

### Analytical methods

Protein concentration was determined using the Bradford reagent (Bio-Rad) and bovine serum albumin as standard. SDS-PAGE was carried out using Precast Polyacrylamide (12%) Mini Gels (Thermo Scientific). Page Blue Protein Stain (Fermentas) was used for SDS-PAGE gels staining.

Glycosylation sites in McoA, McoB and McoG amino acid sequences were predicted using NetNGlyc 1.0 (http://www.cbs.dtu.dk/services/NetNGlyc/) and GPP Prediction Servers (http://comp.chem.nottingham.ac.uk/glyco/index.html).

Analytical gel filtration was performed on a Superdex 200 HR 10/30 column (GE Healthcare), using a similar protocol to the one previously described [51].

### Enzyme activity determination

All chemicals were purchased from Sigma and Invitrogen. McoA, McoB and McoG activity was determined using similar conditions as previously described [12], by measuring the initial oxidation rate (*Δ*Α), during a period of 6 min, of 6.0 mM ABTS at 420 nm and 2.5 mM DMPPDA at 550 nm (pH 5.0). Enzyme kinetics were determined measuring the oxidation rate of, at least, 10 different solutions of ABTS and DMPPDA, in concentrations ranging from 0.125 to 15 mM. The kinetic parameters of the three enzymes were determined by Michaelis-Menten analysis using the Sigma Plot 8.0 Software for Enzyme Kinetics*. V*_*max*_ values were expressed in *Δ*absorbance/min mg protein.

### Effect of pH and temperature on MCO activity

The pH-dependent activity of McoA, McoB and McoG with ABTS (6.0 mM) or DMPPDA (2.5 mM) was measured in McIlvaine's buffer ranging from pH 2.2 to 8.0 as mentioned in the “Enzyme activity determination” section. The temperature-dependent activity of McoB and McoG was measured from 30 to 100°C by end point determination. Reaction mixtures (in closed microcentrifuge tubes) were incubated at different temperatures for 6 min with ABTS (6.0 mM) in 100 mM sodium acetate buffer (pH 5.0). After an incubation step on ice of 5 min, the absorbance of the samples was measured at 420 nm.

### Effect of inhibitors on MCO activity

The activity of McoA, McoB and McoG with 2.5 mM DMPPDA in 100 mM sodium acetate buffer (pH 5.0) was measured at 23°C in the absence and presence of 1.0 mM NaN_3_; 1.0 mM SDS; 50 mM EDTA, CaCl_2_, and MgCl_2_; 100 mM NaCl; different concentrations of CuSO_4_ (0.1, 0.5, 1.0 and 2.5 mM) and 50% methanol, ethanol and acetone.

### Substrate specificity

The activity of McoA, McoB and McoG with the following aromatic compounds was investigated: ADBP, phenol, 2,6-dimethoxyphenol, hydroquinone, vanillin, *p*-coumaric acid, vanillic acid, ferulic acid, syringic acid, and cinnamic acid. Quantification of substrate specificity was achieved through direct measurement of oxygen consumption. For this purpose an Oxytherm (Hansatech Instruments) was used. All reaction mixtures contained 1 mM of substrate and 100 mM sodium acetate buffer (pH 5.0), and reactions were performed at 23°C. One unit of enzyme activity (U) was defined as the amount of enzyme that oxidizes 1 μmol of substrate per min.

### Dye decolorization

Bromocresol purple (440 nm), amido black 10B (600 nm), crystal violet (560 nm), malachite green (600 nm), blue dextran (600 nm) and bromothymol blue (440 nm) were incubated with 100 μg/mL MCO in 100 mM sodium acetate buffer (pH 5.0) at 55°C for 20 h. As a negative control, the different dyes were incubated with an equal volume of 10 mM HEPES buffer (pH 7.0). The degree of dye decolorization was measured 3 and 20 h after the incubation started.

### Fungicide activity of MG and its transformation products

A malachite green solution (100 mg/L) was incubated with McoB or an equal volume of 10 mM HEPES buffer (pH 7.0), for 3 and 20 h, following the conditions mentioned in the “Dye decolorization” methods section. After the 3 h incubation, DMG and MG were mixed with *Aspergillus* complete medium in 24 well agar plates to reach the following concentrations: 0.25 mg/L, 0.5 mg/L, 1 mg/L, 2 mg/L and 4 mg/L. Similarly, after 20 h incubation, complete medium agar plates were prepared with DMG and MG at the following concentrations: 6 mg/L, 10 mg/L and 20 mg/L. DMG and MG toxicity was measured by their ability to inhibit or reduce *A. niger* mycelium radial growth after an incubation period of 24 h. In order to monitor toxicity effects and major growth delays produced by DMG or MG, the mycelium growth was monitored for two weeks.

## Competing interests

The authors declare that there are no competing interests.

## Authors’ contributions

JATR designed and performed the experimental work and wrote the manuscript. WJHB collaborated in the coordination of the research and helped to draft the manuscript. LG designed the study and helped to draft the manuscript. All authors read and approved the submission of the manuscript.
